# Race and virulence characterization of *Puccinia graminis* f. sp. *tritici* in China

**DOI:** 10.1371/journal.pone.0197579

**Published:** 2018-05-24

**Authors:** Tian Ya Li, Yu Chen Ma, Xian Xin Wu, Si Chen, Xiao Feng Xu, Hao Wang, Yuan Yin Cao, Yuan Hu Xuan

**Affiliations:** 1 College of Plant Protection, Shenyang Agricultural University, Shenyang, Liaoning, China; 2 Industrial Crops Institute, Heilongjiang Academy of Agricultural Sciences, Harbin, Heilongjiang, China; Institute for Resistance Research and Stress Tolerance, GERMANY

## Abstract

Wheat stem rust was once the most destructive plant disease, but it has been largely controlled. However, to prevent future problems, the ongoing development of resistant wheat varieties requires knowledge of the changing virulence patterns for *Pgt* virulence of the fungus that causes wheat stem rust and the detection of new races. Surveys were conducted from 2013–2014 to determine the races of the *Pgt* present in China. Low levels of stem rust infections were found in China during this investigation and 11 *Puccinia graminis* f. sp. *tritici* (*Pgt*) samples were obtained. In addition, 22 *Pgt* samples collected from the alternate host (*Berberis*) were obtained and have been reported for the first time. Fifty-three isolates were obtained from all samples. Four race groups, including 13 physiological races, were identified. They included the most prevalent races, 34C3MTGQM and 34C6MRGQM, with 13.2% predominance, followed by 34C0MRGQM at 11.3%. Six new races that were virulent against the resistance genes, *Sr5* + *Sr11*, were found for the first time in China, namely 34C0MRGQM, 34C3MTGQM, 34C3MKGQM, 34C3MKGSM, 34C6MTGSM, and 34C6MRGQM, with a predominance of 11.3, 13.2, 9.4, 9.4, 1.9, and 13.2%, respectively. Most of the genes studied were ineffective against one or more of the tested isolates, except *Sr9e*, *Sr21*, *Sr26*, *Sr31*, *Sr33*, *Sr38*, *Sr47*, and *SrTt3*. Genes *Sr35*, *SrTmp*, *Sr30*, *Sr37*, *Sr17*, and *Sr36* were effective in 92.5, 86.8, 84.9, 84.9, 79.3, and 77.4% of the tested isolates, respectively. In contrast, all of the isolates were virulent against *Sr6*, *Sr7b*, *Sr9a*, *Sr9b*, *Sr9d*, *Sr9g*, and *SrMcN*. Our results indicate that remarkable differences exist among the categories of the races in this study (i.e., their known virulence gene spectra) and the *Pgt* races reported previously. In addition, the sexual cycle of *Pgt* may contribute to its diversity in China.

## Introduction

Wheat stem rust caused by *Puccinia graminis* Pers. f. sp. *tritici* Eriks. and E. Henn. (*Pgt*) is a major disease of wheat. Heavy epidemics occurred frequently from the 1920s until the 1960s, causing huge yield losses. In recent years, because of the cultivation and utilization of resistant cultivars of wheat, and changes in the cultivation system in China after 1980, wheat stem rust now only occurs sporadically under natural conditions in the Yangtze River area [1]. However, it is undeniable that this disease had devastated wheat production in China and other parts of the world. Exploring resistance genes and growing resistant cultivars are effective measures for controlling this disease because of low cost, effectiveness, and environmental friendliness [2]. However, the changing virulence patterns and the evolution of fungal races constitute a threat to most wheat germplasm resources. For example, the new races, TTKSK (known as Ug99), TKTTF, and TTTTF, and their variants, threaten wheat production worldwide [3–5].

Maintaining dynamic information on pathogens is the basis for breeding disease resistance. Although wheat stem rust has not been common in recent years, understanding the population structure, virulence diversity, and distribution of *Pgt* at various times and in different locations is essential for selecting effective resistance genes to control this disease [6]. Furthermore, work on detecting and identifying races of *Pgt* has not stopped. Particularly, the emergence and rapid propagation of Ug99 and its variants have attracted increasing attention worldwide, and 28 trapping plots or nurseries have been established yearly in China under the unified arrangement of the wheat rust and powdery mildew cooperative group [7]. Simultaneously, wheat breeding and disease researchers have focused on the investigation and collection of *Pgt* to identify and analyze wheat stem rust. Particularly, aecia from the wheat stem rust alternate host (*Berberis*) were collected from 2013–2014. The objective of the present study was to analyze the pathotypes and characterization of *Pgt* races.

## Materials and methods

### Ethic statement

Samples in this study were obtained from public areas that no specific permissions were required for these locations. Fields sites are public access and *P*. *graminis* f. sp. *tritici* is not an endangered or protected species.

### Collection of wheat stem rust samples

**Fig 1:**
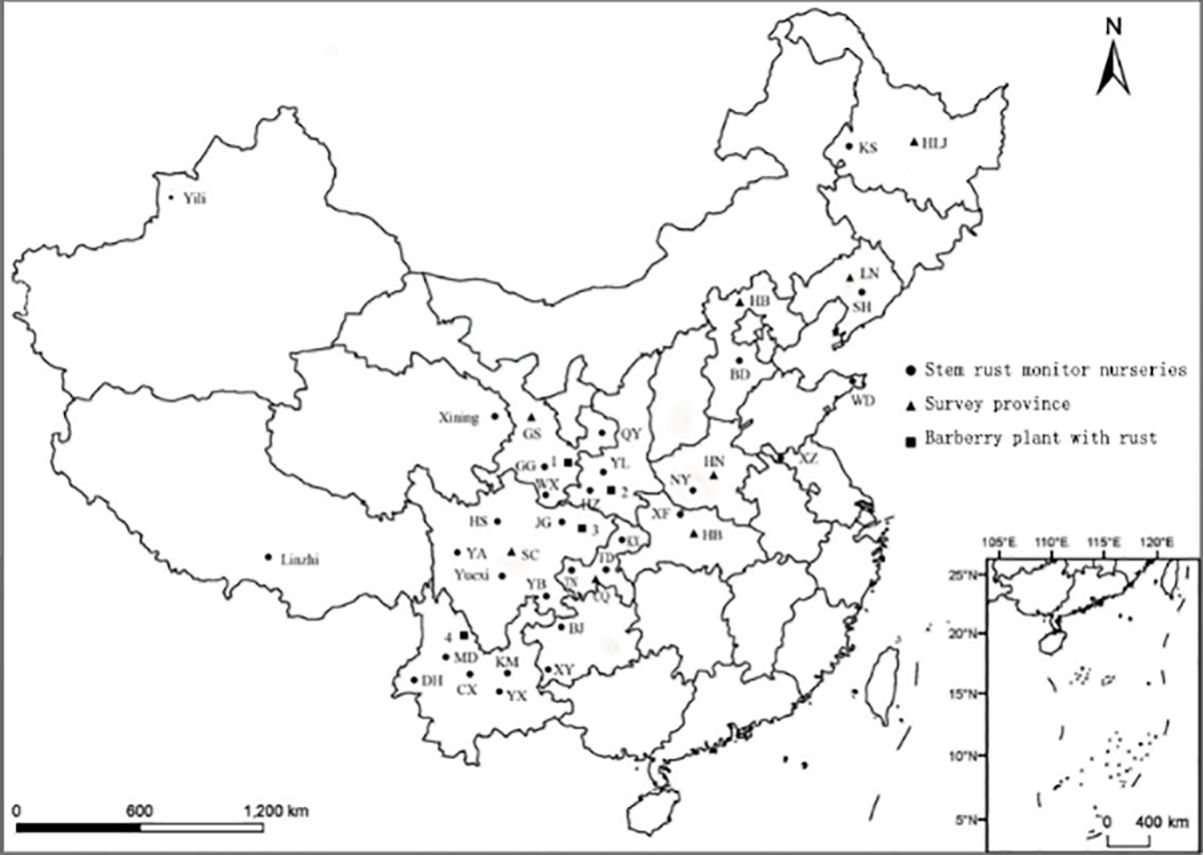
Regions for the detection and investigation of Pgt in 2013–2014. Note: ▲: HLJ: Heilongjiang, LN: Liaoning, HB: Hebei, HN: Henan, HB: Hubei, CQ: Chongqing, GS: Gansu, SC: Sichuan, YN: Yunnan. ●: KS: Keshan, SY: Shenyang, BD: Baoding, WD: Wendeng, HZ: Hanzhong, YL: Yangling, GG: Gangu, QY: Qingyang, WX: Wenxian, HS: Heishui, YA: Yaan, YB: Yibing, JG: Jiange, KX: Kaixian, FD: Fengdu, TN: Tongnan, YX: Yuxi, MD: Midu, KM: Kunming, ST: Shaotong, DH: Dehong, XY: Xingyi, BJ: Bijie, XF: Xiangfan, XZ: Xuzhou. ■: 1: Southeastern Gansu Province 2: Southwestern Shannxi Province 3: Northeastern Sichuan Province 4: Northwestern Yunnan Province.

In 2013–2014, eleven samples of rust-infected stems were collected from wheat fields in Hubei, Heilongjiang, Liaoning, Guizhou, Sichuan, Yunnan, and Gansu provinces (Fig 1), and 22 samples previously collected from the alternate host, *Berberis*, alternate host for many *Puccinia* spp. including *graminis* and *striiformis*, were isolated and identified.

### Isolation and multiplication of spores

Seedlings of the universally rust-susceptible wheat variety, Little Club (LC), were raised in 12-cm diameter porcelain pots for the propagation of strains. Infected samples were removed from the refrigerator (4°C), and single pustules from the wheat stem rust samples were isolated in order to inoculate leaves of LC collected from seven-day-old seedlings or seedlings with fully expanded primary and secondary leaves. We followed the procedure of Huang [8]. LC leaves were cut into small pieces (6–7 cm long) and were placed between two pieces of filter paper kept in 15-cm petri dishes. The two ends of each leaf segment were pressed with glass (length 120 mm, width 10 mm, thickness 3 mm) and 40 mg·L^-1^ 6-benzylaminopurine (BA) was added as a preservative solution to soak the filters. The uredia were scraped from the rust-infected stem samples using flat toothpicks and were then sprayed on the abaxial surface of the lamina. Each leaf was inoculated at two spots by daubing gently, preventing scratching of the foliage. Thereafter, the inoculated leaves were moistened with 0.05% Tween 20 using an atomizer, and were placed in an incubation chamber for 20 h in the dark at 16–20°C. Subsequently, they were transferred from the dew chamber to glass compartments in an artificial culture room, where the conditions were controlled with a 14 h:10 h (light:dark) photoperiod and temperature of 18–22 ± 1°C. When the uredinia (diameter of 0.5–0.7 mm) appeared after 5–6 days, single pustules were isolated and used for re-inoculation. The spore multiplication procedure was repeated 2–3 times until sufficient numbers of spores were produced for experimentation. Twenty-three and 30 single pustules were identified from 11 samples obtained from wheat and 22 samples obtained from *Berberis*, respectively. All materials used in the experimental set-up had been sterilized.

### Inoculation of differential lines and designation of races

Differential wheat were planted in 12-cm diameter porcelain pots [9]. Each pot contained four genotypes (5–6 seeds per genotype) separated from each other. The susceptible wheat variety, LC, was used as the control to ascertain the viability of the spores. The leaves of seven-day-old seedlings were moistened with 0.05% Tween 20 using an atomizer. Thereafter, purified single-pustule isolates were sprayed/inoculated evenly onto the back of the leaves with flat toothpicks and then moistened again with 0.05% Tween 20. After inoculation, the plants were placed in a dew chamber with 16–20 h of darkness at 18–22°C. Then, they were transferred to a greenhouse at a temperature of 18–22 ± 1°C and relative humidity (RH) of 65–75%. The infections were assessed 14 days after inoculation using the 0–4 Infection Types (ITs) scale, as described by Stakman et al. [10]. The infections were grouped into two types, where values of 0, 1, 1+, 2, and 2+ were considered low infection types (indicating resistance) and values of 3-, 3, 3+, and 4 were considered high infection types (indicating susceptibility). Race names were designated according to the methods described by Li et al. [9]. In this study, the isolates were purified and assessed in three replicates to obtain uniform data regarding infection type.

### Determination of virulence frequency of races

Forty-six single *Sr* gene lines were used to test the virulence frequency of the races, including 20 North American single genes (*Sr5*, *Sr21*, *Sr9e*, *Sr7b*, *Sr11*, *Sr6*, *Sr8a*, *Sr9g*, *Sr36*, *Sr9b*, *Sr30*, *Sr17*, *Sr9a*, *Sr9d*, *Sr10*, *SrTmp*, *Sr24*, *Sr31*, *Sr38*, and *SrMcN*) and 26 additional single genes provided by the Minnesota Cereal Disease Laboratory (*Sr9f*, *Sr12*, *Sr13*, *Sr14*, *Sr15*, *Sr16*, *Sr18*, *Sr19*, *Sr20*, *Sr22*, *Sr23*, *Sr25*, *Sr26*, *Sr27*, *Sr28*, *Sr29*, *Sr32*, *Sr33*, *Sr34*, *Sr35*, *Sr37*, *Sr39*, *Sr47*, *SrTt3*, *SrGT*, and *SrWld-1*). The inoculation and assessment methods followed the procedures mentioned earlier.

## Results

### Occurrence and frequency of *Pgt* races

From the twenty-three single pustules obtained from the 11 samples, we identified six races, 21C3CTHTM, 21C3CTQSC, 21C3CTTSC, 21C3CTTTM, 34C0MKGQM, and 34C0MRGSM that can be included in the race groups, 21C3 and 34C0. Races 21C3CTQSC, 21C3CTTSC, 21C3CTTTM, and 34C0MRGSM are new physiological races. To the best of our knowledge, this is the first report of the virulence of 34C0MRGSM to *Sr5* + *Sr11* in China. Race 21C3CTTTM was virulent to the 20 North American single-gene differentials, except *Sr5*, *Sr9e*, *Sr31*, and *Sr38* (Table 1). Thirty single pustules were identified from the 22 samples obtained from *Berberis*. Three race groups, 34C0, 34C6, and 34C3, including seven physiological races, were identified. Six of the seven races are novel and virulent against *Sr5* + *Sr11*, namely 34C0MRGQM, 34C6MTGSM, 34C6MRGQM, 34C3MTGQM, 34C3MKGQM, and 34C3MKGSM, whereas the other race (34C0MTGSM) has been previously identified.

**Table 1:** Infection types of identified *P*. *graminis* f. sp. *tritici* races collected from 2013–2014 in China toward differential wheat cultivars.

Differentials	*Sr* genes	R1	R2	R3	R4	R5	R6	R7	R8	R9	R10	R11	R12	R13^a^
ISr5-Ra	*5*	1^b^	0	1	0	4	4	4	4	4	3	4	3+	4
CnS_T_mono_deriv	*21*	2	1+	1-	1+	2	;1 =	1+	2	2	1	2	1+	1+
Vernstine	*9e*	1-	;	;1 =	1-	1	;	;1-	1	;1	;1	1+	1	1
ISr7b-Ra	*7b*	4	4	4	4	4	4	4	4	4	3	4	4	4
ISr11-Ra	*11*	3	4	3	4	0	4	4	4	4	3	1	;	4
ISr6-Ra	*6*	3	4	4	4	4	4	4	4	4	3	3	4	3
ISr8a-Ra	*8a*	4	3	4	4	4	1	1+	3	2	3	3	4	4
CnSr9g	*9g*	4	4	4	4	4	4	4	4	4	4	4	4	4
W2691SrTt-1	*36*	0	4	4	4	0	0	0	1	1	0	0	0	1
W2691Sr9b	*9b*	4	4	3	4	3	3	4	4	4	4	4	4	4
BtSr30Wst	*30*	1	2	4	4	1	;	1	1	2	1	1	;	1
Combination VII	*17+13*	4	2	3	4	0	0	0	;	0	;	0	;	;
ISr9a-Ra	*9a*	4	4	3	3	4	4	4	4	4	4	4	4	4
ISr9d-Ra	*9d*	4	4	4	4	4	4	4	4	4	4	4	4	4
W2691Sr10	*10*	4	4	4	4	1+N	1+N	3	4	1+N	3	;	3C	;
CnsSrTmp	*Tmp*	3	1C	1-	3	0	1N	1+	;	1N	0	0	;	;
LcSr24Ag	*24*	3C	3	;1 =	3	3	3	4	4	4	4	4	3	4
Sr31/6*LMPG	*31*	1C	;1-	1-	1	1	;	1	1	1	1	1	1	1
Trident	*38*	;	0	1N	;	1	;	;1-	;	1N	;1-	;	;	;1-
McNair 701	McN	4	4	4	4	4	4	4	4	4	4	4	4	4
Reliance	*5*, *16*, *18*,*20*	0	0	0	0	4	4	4	4	4	4	4	4	3
Vernal	*9e*	;1 =	;1 =	;	;1	;1-	;	;1	1	1	1	1	;1	1
Einkorn	*21*	1	;	;1 =	1	;1 =	;	;1-	1	;1	1	;1-	;1-	;1
Mianzi 52	*17*, *+*?^*C*^	4	4	4	4	;	0	0	;	0	0	0	0	;
Mini 2761	*5*, *+*?	0	0	0	0	;	0	0	0	0	0	0	0	;
Orofen	*5*, *6*, *+*?	0	0	0	;	0	;	1	;	2	2	3	4	4
Rulofen	*6*, *30*, *+*?	0	;	0	0	0	0	0	0	0	0	0	0	0
Little Club	*LC*	4	4	4	4	4	4	4	4	4	4	4	4	4

^a^ R1: 21C3CTHTM; R2: 21C3CTQSM; R3:21C3CTTSC; R4:21C3CTTTM; R5: 34C0MKGQM; R6: 34C0MRGQM; R7:34C0MRGSM

R8: 34C0MTGSM; R9: 34C6MRGQM; R10: 34C6MTGSM; R11: 34C3MKGQM; R12:34C3MKGSM; R13: 34C3MTGQM

^b^ Infection types (ITs): are based on a 0-to-4 scale where ITs of 0, 1, or 2 are indicative of a resistant (low) response and ITs of 3 or 4 are indicative of a susceptible (high) response; Symbols + and–indicate slightly larger and smaller pustule sizes, respectively; N was used for cases with significant necrosis [10].

^C^ Symbol ‘+?’ means the cultivar carry other unknown resistance gene.

Among the 13 races identified from the 53 isolates, the most predominant races were 34C3MTGQM and 34C6MRGQM, each with a frequency of 13.2%. The next predominant race was 34C0MRGQM, with a frequency of 11.3%. (Table 2).

**Table 2:** Races of *P*. *gramins* f. sp. *tritici* in China from 2013–2014.

Race	Host	Province (No. of isolates)	Frequency (%)
21C3CTHTM	Wheat	Liaoning (3)	5.7
21C3CTQSM	Wheat	Guizhou (1); Yunnan (3)	7.5
21C3CTTSC	Wheat	Guizhou (4)	7.5
21C3CTTTM	Wheat	Guizhou (2); Hubei (2)	7.5
34C0MRGSM	Wheat	Sichuan (1); Hubei (1)	3.8
34C0MKGQM	Wheat, Barberry	Sichuan (1); Heilongjiang (1)	3.8
34C0MRGQM	Wheat, Barberry	Heilongjiang (5); Gansu (1)	11.3
34C0MTGSM	Barberry	Gansu (3)	5.7
34C6MRGQM	Barberry	Shaanxi (1); Gansu (6)	13.2
34C6MTGSM	Barberry	Gansu (1)	1.9
34C3MKGQM	Barberry	Shaanxi (2); Gansu (4)	9.4
34C3MKGSM	Barberry	Shaanxi (1); Gansu (3)	9.4
34C3MTGQM	Barberry	Shaanxi (2); Gansu (5)	13.2

### Virulence of *Pgt* towards 46 single-gene differentials

The virulence frequencies of the 53 isolates against *Sr6*, *Sr7b*, *Sr9a*, *Sr9b*, *Sr9d*, *Sr9g*, and *SrMcN* were 100%, and those against *Sr5*, *Sr8a*, *Sr9f*, *Sr12*, *Sr13*, *Sr15*, *Sr16*, *Sr18*, *Sr20*, *Sr27*, *Sr28*, *Sr29*, *Sr32*, and *Sr39* were also high, with frequencies of 58.5–96.2%. However, the virulence frequencies toward *Sr10*, *Sr14*, *SrGT*, *Sr25*, *Sr19*, *Sr22*, *Sr23*, and *SrWld-1* were relatively low, with frequencies of 49.1, 47.2, 45.3, 43.4, 41.5, 37.7, 35.8, and 33.9%, respectively (Table 3).

**Table 3:** Virulence frequencies of races of *P*. *graminis* f. sp. *tritici* collected from China during 2013–2014.

*Sr* gene	No. of virulent isolates	Virulence frequency (%)	*Sr* gene	No. of virulent isolates	Virulence frequency (%)
*5*	38	71.7	*23*	19	35.8
*6*	53	100	*24*	45	84.9
*7b*	53	100	*25*	23	43.4
*8a*	38	71.7	*26*	0	0
*9a*	53	100	*27*	41	77.4
*9b*	53	100	*28*	43	81.1
*9d*	53	100	*29*	39	73.6
*9f*	49	92.5	*30*	8	15.1
*9g*	53	100	*31*	0	0
*9e*	0	0	*32*	43	81.1
*10*	26	49.1	*33*	0	0
*11*	41	77.4	*34*	31	58.5
*12*	44	84.9	*35*	4	7.5
*13*	33	62.3	*36*	12	22.6
*14*	24	47.2	*37*	8	15.1
*15*	43	81.1	*38*	0	0
*16*	51	96.2	*39*	45	84.9
*17*	11^a^	20.7	*47*	0	0
*18*	37	69.8	*Tt3*	0	0
*19*	22	41.5	*Tmp*	7	13.2
*20*	41	77.3	*GT*	24	45.3
*21*	0	0	*Wld-1*	18	33.9
*22*	20	37.7	*McN*	53	100

However, the resistance genes, *Sr9e*, *Sr21*, *Sr26*, *Sr31*, *Sr33*, *Sr38*, *Sr47*, and *SrTt3* were effective against all of the 53 isolates tested. Genes *Sr35*, *SrTmp*, *Sr30*, *Sr37*, *Sr17*, and *Sr36* were effective against most of the isolates tested, with a resistance frequency of 92.5, 86.8, 84.9, 84.9, 79.3, and 77.4%, respectively.

The virulence information of 13 physiological races against 46 genes revealed that the races, 21C3CTTTM, 21C3CTTSC, and 21C3CTQSM, included in the 21C3 race group and the race, MKGSM, in the 34C3 race group have broader spectra than do the other races. Race 21C3CTTTM and 34C3MKGSM have the broadest spectra and rendered 34 of 46 stem rust resistant genes ineffective. Race 21C3CTTSC and 21C3CTQSM have second broadest spectra, which demonstrated virulence against 31 of 46 stem rust resistance genes (Table 4).

**Table 4:** Effective/ ineffective *Sr* genes against 13 races of *P*. *graminis* f. sp. *tritici*.

Race	Effective *Sr* genes	Ineffective *Sr* genes
21C3CTHTM	*5*, *9e*, *14*, *19*, *21*, *22*, *23*, *25*, *26*, *28*, *29*, *30*, *31*, *33*, *35*, *36*, *37*, *38*, *47*, *Tt3*	*6*, *7b*, *8a*, *9g*, *9a*, *9b*, *9d*, *9f*, *9g*, *10*, *11*, *12*, *13*, *15*, *16*, *17*, *18*, *20*, *24*, *27*, *32*, *34*, *39*, *Tmp*, *GT*, *Wld-1*, *McN*
21C3CTQSM	*5*, *9e*, *17*, *19*, *20*, *21*, *26*, *27*, *30*, *31*, *33*, *34*, *35*, *37*, *38*, *39*, *47*, *Tmp*, *Tt3*	*6*, *7b*, *8a*, *9a*, *9b*, *9d*, *9f*, *9g*, *10*, *11*, *12*, *13*, *14*, *15*, *16*, *18*, *22*, *23*, *24*, *25*, *28*, *29*, *32*, *36*, *GT*, *Wld-1*, *McN*
21C3CTTSC	*5*, *9e*, *19*, *20*, *21*, *22*, *24*, *26*, *27*, *29*, *31*, *32*, *33*, *37*, *38*, *47*, *Tmp*, *Tt3*,	*6*, *7b*, *8a*, *9a*, *9b*, *9d*, *9f*, *9g*, *10*, *11*, *12*, *13*, *14*, *15*, *16*, *17*, *18*, *23*, *25*, *28*, *30*, *34*, *35*, *36*, *39*, *GT*, *Wld-1*, *McN*
21C3CTTTM	*5*, *9e*, *14*, *19*, *21*, *22*, *23*, *26*, *27*, *31*, *33*, *35*, *37*, *38*, *39*, *47*, *GT*, *Tt3*	*6*, *7b*, *8a*, *9a*, *9b*, *9d*, *9f*, *9g*, *10*, *11*, *12*, *13*, *15*, *16*, *17*, *18*, *20*, *24*, *25*, *28*, *29*, *30*, *32*, *34*, *36*, *Tmp*, *Wld-1*, *McN*
34C0MKGQM	*9e*, *10*, *11*, *13*, *14*, *17*, *18*, *19*, *20*, *21*, *23*, *25*, *26*, *30*, *31*, *32*, *33*, *34*, *35*, *36*, *37*, *38*, *47*, *Tmp*, *Tt3*, *Wld-1*	*5*, *6*, *7b*, *8a*, *9a*, *9b*, *9d*, *9f*, *9g*, *12*, *15*, *16*, *22*, *24*, *27*, *28*, *29*, *39*, *GT*, *McN*
34C0MRGQM	*8a*, *9e*, *10*, *13*, *14*, *17*, *19*, *21*, *22*, *23*, *26*, *27*, *29*, *30*, *31*, *33*, *35*, *36*, *37*, *38*, *47*, *Tmp*, *Tt3*, *GT*, *Wld-1*	*5*, *6*, *7b*, *9a*, *9b*, *9d*, *9f*, *9g*, *11*, *12*, *15*, *16*, *18*, *20*, *24*, *25*, *28*, *32*, *34*, *39*, *McN*
34C0MRGSM	*8a*, *9e*, *17*, *19*, *21*, *22*, *23*, *26*, *30*, *31*, *32*, *33*, *35*, *36*, *37*, *38*, *47*, *Tmp*, *Tt3*	*5*, *6*, *7b*, *9a*, *9b*, *9d*, *9f*, *9g*, *10*, *11*, *12*, *13*, *14*, *15*, *16*, *18*, *20*, *24*, *25*, *27*, *28*, *29*, *34*, *39*, *GT*, *Wld-1*, *McN*
34C0MTGSM	*9e*, *17*, *21*, *26*, *30*, *31*, *33*, *35*, *36*, *37*, *38*, *47*, *GT*, *Tmp*, *Tt3*, *Wld-1*	*5*, *6*, *7b*, *8a*, *9a*, *9b*, *9d*, *9f*, *9g*, *10*, *11*, *12*, *13*, *14*, *15*, *16*, *18*, *19*, *20*, *22*, *23*, *24*, *25*, *27*, *28*, *29*, *32*, *34*, *39*, *McN*
34C6MTGSM	*9e*, *12*, *17*, *19*, *21*, *22*, *23*, *26*, *27*, *30*, *31*, *33*, *34*, *35*, *36*, *38*, *47*, *GT*, *Tmp*, *Tt3*, *Wld-1*	*5*, *6*, *7b*, *8a*, *9a*, *9b*, *9d*, *9f*, *9g*, *10*, *11*, *13*, *14*, *15*, *16*, *18*, *20*, *24*, *25*, *28*, *29*, *32*, *37*, *39*, *McN*
34C6MRGQM	*8a*, *9e*, *10*, *14*, *17*, *21*, *23*, *26*, *30*, *31*, *32*, *33*, *35*, *36*, *38*, *47*, *Tmp*, *Tt3*, *Wld-1*	*5*, *6*, *7b*, *9a*, *9b*, *9d*, *9f*, *9g*, *11*, *12*, *13*, *15*, *16*, *18*, *19*, *20*, *22*, *24*, *25*, *27*, *28*, *29*, *34*, *37*, *39*, *GT*, *McN*
34C3MKGQM	*9e*, *10*, *11*, *13*, *14*, *15*, *17*, *18*, *20*, *21*, *22*, *25*, *26*, *30*, *31*, *33*, *34*, *35*, *36*, *37*, *38*, *47*, *GT*, *Tmp*, *Tt3*, *Wld-1*	*5*, *6*, *7b*, *8a*, *9a*, *9b*, *9d*, *9f*, *9g*, *12*, *16*, *19*, *23*, *24*, *27*, *28*, *29*, *32*, *39*, *McN*
34C3MKGSM	*9e*, *11*, *17*, *21*, *26*, *30*, *31*, *33*, *35*, *36*, *37*, *38*, *Tmp*, *Tt3*, *47*,	*5*, *6*, *7b*, *8a*, *9a*, *9b*, *9d*, *9f*, *9g*, *10*, *12*, *13*, *14*, *15*, *16*, *18*, *19*, *20*, *22*, *23*, *24*, *25*, *27*, *28*, *29*, *32*, *34*, *39*, *GT*, *Wld-1*, *McN*
34C3MTGQM	*9e*, *10*, *15*, *17*, *19*, *21*, *25*, *26*, *27*, *29*, *30*, *31*, *33*, *35*, *36*, *37*, *38*, *47*, *Tmp*, *Tt3*, *GT*, *dp-2*, *Wld-1*	*5*, *6*, *7b*, *8a*, *9a*, *9b*, *9d*, *9f*, *9g*, *11*, *12*, *13*, *14*, *16*, *18*, *20*, *22*, *23*, *24*, *28*, *32*, *34*, *39*, *McN*

## Discussion

Due to the potential threat of new emerging races of *Pgt* such as Ug99 and their potential threat to global wheat production, this study aimed to identifying and characterising *Pgt* races in China from 2013–2014. The results revealed race groups that had been documented earlier, such as 34C1 and 34C2, and rare race groups (e.g., 34C4 and 116), all of which were not detected in the present study. In the 2013–2014 study, race groups 21C3 and 34C3 were predominant in China [11, 12]. Previous studies suggested that this observation was related to the strong competitiveness of this pathogen [13, 14]. Its wide spectrum of virulence may be another reason [11]. This was confirmed in our study by testing the virulence of 53 different strains against 46 resistance genes. For instance, race CTTTM, which has the widest virulence spectrum in group 21C3, was virulent to 33 of the 46 resistance genes, including *Sr17*, *Sr24*, *SrTmp*, and *Sr36*. Only 15 resistance genes, specifically *Sr5*, *Sr9e*, *Sr14*, *Sr19*, *Sr22*, *Sr23*, *Sr26*, *Sr27*, *Sr31*, *Sr35*, *Sr37*, *Sr38*, *Sr47*, *SrGT*, and *SrTt3*, were found to be effective against CTTTM. Race group 21C3 was dominant, occurring with high frequency, and significant changes in its population structure have occurred. For example, races CTHTM, CTQSM, CTTSC, and CTTTM are new to the 21C3 group.

*Berberis* is the alternate host of *Pgt* and plays a vital role throughout the sexual life cycle of *Pgt* [15]. Sexual reproduction of *Pgt* is completed on the leaves of *Berberis*, resulting in the diversity of *Pgt* races [16, 17]. Since the 1950s, wheat stem rust has been effectively controlled by using highly resistant winter/spring wheat cultivars and eradicating the common barberry in the United States [18, 19], whereas in China, insufficient research was carried out to determine whether sexual reproduction could lead to diversity of the *Pgt* population. However, after *Berberis* was shown to be the alternate host of *P*. *striiformis* f. sp. *tritici* (*Pst*) in 2010 [16], this plant Genus was found to be distributed widely in China, and it is infected by *Pst* under natural conditions. Researchers believed that the diversity of *Pst* would be contributed through the sexual cycle of the fungus on Berberis [20]. Our results are consistent with this hypothesis; for example, the races identified from isolates that were collected from aecia are all new races, except 34C0MKGQM. Race group 34C3 was first discovered in 1976 and threatened the Orofen wheat cultivar and its progeny planted in more than million hectares. This caused great fear, but fortunately, owing to a series of timely emergency measures taken by researchers, the occurrence of race group 34C3 remained low from 1983 to 1987, and it has not been detected since 1987 [8]. In 1990, three races, 34C3MKGQM, 34C3MKGSM, and 34C3MTGQM, were identified for the first time in China by using the North American stem rust nomenclature system of *Pgt* [21]. Race MKGSM, having the widest virulence spectrum, possessed virulence to the remaining 30 resistance genes, except *Sr9e*, *Sr11*, *Sr17*, *Sr26*, *Sr30*, *Sr31*, *Sr33*, *Sr35*, *Sr36*, *Sr37*, *Sr38*, *Sr47*, *SrTmp*, and *SrTt3*. In addition, MKGSM and MTGQM had wide virulence spectra.

In our study, it was evident that the majority of the resistance genes were ineffective against most of the isolates according to the information and frequency of virulence. Among the 46 single-genes, *Sr9e*, *Sr21*, *Sr26*, *Sr31*, *Sr38*, *Sr47*, and *SrTt3* were effective against all of the isolates and these 7 currently effective resistance genes should be used in breeding programs to ensure that Chinese wheat cultivars are resistant to the new races, while *Sr17*, *Sr30*, *Sr33*, *Sr35*, *Sr36*, *Sr37*, and *SrTmp* were effective against most of the isolates. Similar results were reported by Han et al. and Yao et al. [11, 22]. However, except for the 14 resistance genes mentioned directly above, the resistance genes tested was ineffective against one or more isolates. More than 58.5% of the isolates identified were virulent to *Sr5*, *Sr6*, *Sr7b*, *Sr13*, *Sr24*, *Sr32*, and *Sr34*. However, these resistance genes exist widely in wheat varieties of China [23, 24]. Notably, the MR races (e.g., 34C0MRGSM) and MT races (e.g., 34C6MTGSM) were first discovered in China. They are virulent against *Sr5* + *Sr11*, which are two of the most commonly used resistance genes. This is a threat to wheat production [25]; therefore, urgent attention is necessary to address this situation.

## References

[1] Cao YY, Yao P, Bi YQ, Yang JX. Discovery and verification of an important inoculum source for *Puccinia graminis* f. sp. *tritici* in China. Plant Protection. 2001; 28: 294–298.

[2] Goutam U, Kukreja S, Yadav R, Salaria N, Thakur K, Goya AK. Recent trends and perspectives of molecular markers against fungal diseases in wheat. Frontiers in Micbio. 2015; 6: 861 doi: 10.3389/fmicb.2015.00861 eCollection 2015. 26379639 10.3389/fmicb.2015.00861 PMC4548237

[3] Bhattacharya S. Deadly new wheat disease threatens Europe’s crops. Nature. 2017; 542: 145–146. doi: 10.1038/nature.2017.21424 28179687 10.1038/nature.2017.21424

[4] Olivera P, Newcomb M, Szabo LJ, Rouse M, Johnson J, Gale S, et al Phenotypic and genotypic characterization of race TKTTF of *Puccinia graminis* f. sp. *tritici* that caused a wheat stem rust Epidemic in Southern Ethiopia in 2013–14. Phytopathology. 2015; 105: 917–928. doi: 10.1094/PHYTO-11-14-0302-FI 25775107 10.1094/PHYTO-11-14-0302-FI

[5] Pretorius ZA, Singh RP, Wagoire WW, Payne TS. Detection of virulence to wheat stem rust resistance gene *Sr31* in *Puccinia graminis* f. sp. *tritici* in Uganda. Plant Dis. 2000; 84: 203 doi: 10.1094/PDIS.2000.84.2.203B 10.1094/PDIS.2000.84.2.203B 30841334

[6] Abebe T, Woldeab G, Dawit W. Distribution and physiologic races of wheat stem rust in Tigray, Ethiopia. J Plant Pathol Microbiol. 2012; 3: 1–6. doi: 10.4172/2157-7471.1000142

[7] FAO. 2017. Spread of damaging wheat rust continues: new races found in Europe, Africa, Central Asia. 3 February. Available at http://www.fao.org/news/story/en/item/469467/icode/.

[8] Huang ZT, Yao P, Wu YS. Racial identification of wheat stem rust in 1985. Acta Phytopathol Sinica. 1988; 15: 29–32.

[9] Li TY, Wu XX, Xu XF, Wang WL, Cao YY. Postulation of seedling stem rust resistance genes of Yunnan wheat cultivars in China. Plant Prot Sci. 2016; 4: 242–249. doi: 10.17221/137/2015-PPS

[10] Stakman EC, Stewart DM, Loegering WQ. Identification of physiologic races of *Puccinia graminis* var. *tritici*. US Department of Agric, 1962, ARSE-617, p53.

[11] Han JD, Cao YY, Sun ZG. Race dynamics of *Puccinia graminis* f. sp. *tritici* in China and the virulence of CIMMYT wheat germplasm resistant to Ug99. Journal of Triticeae Crops. 2010; 30: 163–166.

[12] Yao P, Cao YY, Liu WZ, Wu YS. Race dynamics analysis of *Puccinia graminis* f. sp. *tritici* in China in 1994, J Shenyang Agric Univ. 1996; 27: 263–268.

[13] Cao YY, Yao P, Huang ZT, Zhu GQ, Wu YS. Competitive ability among races of *Puccinia graminis* f. sp. *tritici* in mixtures. Acta Phytophylacica Sinica. 1996; 23:45–50.

[14] Huang ZT, Cao YY, Yao P, Wu YS. Studies on the relative survival ability of different races of *Puccinia graminis* f. sp. *tritici* in mixtures. Acta Phytopathol Sinica. 1991; 21: 65–71.

[15] de Bary A. Neue Untersuchungen ber die Uredineen, insbesondere die Entwicklung der *Puccinia graminis* und den Zusammenhang derselben mit Aecidium *Berberidis*. 1866. Monatsber. K. Preuss. Akad. Wiss. Berlin.

[16] Jin Y, Szabo LJ, Carson M. Century-old mystery of *Puccinia striiformis* life history solved with the identification of *Berberis* as an alternate host. Phytopathology. 2010; 100: 432–435. doi: 10.1094/PHYTO-100-5-0432 20373963 10.1094/PHYTO-100-5-0432

[17] Roelfs AP, Groth VJ. A comparison of virulence phenotypes in wheat stem rust populations reproducing sexually and asexually. Phytopathology. 1980; 70: 855–862

[18] Roelfs AP. Effects of Barberry eradication on stem rust in the United States. Plant Dis. 1982; 66: 177–181.

[19] Kolmer JA, Jin Y, Long DL. Wheat leaf and stem rust in the United States. Australian Journal of Agricultural Research. 2007; 58: 631–638. doi: 10.1071/AR07057

[20] Zhao J, Wang L, Wang ZY, Chen XM, Zhang HC, Yao J, et al Identification of Eighteen Berberis Species as Alternate hosts of *Puccinia striiformis* f. sp. *tritici* and virulence variation in the pathogen isolates from natural infection of barberry plants in China. Phytopathology. 2013; 103: 927–933. doi: 10.1094/PHYTO-09-12-0249-R 23514262 10.1094/PHYTO-09-12-0249-R

[21] Yao P, Cao YY, Wu YS. Race identification of *Puccinia graminis* f. sp. *tritici* in China in 1990. Acta Phytophylacica Sinica. 1993; 20: 65–70.

[22] Yao P, Cao YY, Zhang SS. Race dynamics analysis of *Puccinia graminis* f. sp. *tritici* in China. Plant Prot. 1998; 24: 3–6.

[23] Chen WQ, Wang JX. Genes for leaf and stem rust resistance in 76 wheat genetic resource, Acta Agronomica Sinica.1997; 23: 655–663.

[24] Qiu YC, Zhang SS, Liu YL. Postulation of resistance genes to stem rust in 120 wheat cultivars in North China. J Triticeae Crops. 1999; 30: 231–234.

[25] Cao YY, Yao P, Liu WZ Wu YS. Pathogenic spectrum analysis of 21C3CTR of *Puccinia graminis* f. sp. *tritici* in China. J Shenyang Agric. 1996; 27: 26–30.

